# Periodontal Tissue Healing After Autologous Tooth Transplantation: A Retrospective Analysis of Case Series

**DOI:** 10.3290/j.ohpd.b2403411

**Published:** 2021-12-08

**Authors:** Yohei Kamata, Tomoko Shimizu, Akira Tsunoda, Toshiyuki Tamura, Motohiro Komaki, Toshiro Kodama

**Affiliations:** a Dental Surgeon, Department of Highly Advanced Stomatology, Yokohama Clinic, Kanagawa Dental University, Kanagawa, Yokohama, Japan. Study idea and hypothesis; assessed the feasibility of the experimental design; performed the experiments and wrote the manuscript.; b Dental Surgeon, Department of Highly Advanced Stomatology, Yokohama Clinic, Kanagawa Dental University, Kanagawa, Yokohama, Japan. Study idea and hypothesis; recruited the participants and conducted the follow-up evaluations.; c Associate Professor, Department of Dental Hygiene, Kanagawa Dental University Junior College, Yokosuka, Japan. Assessed the feasibility of the experimental design; recruited the participants and conducted the follow-up evaluations.; d Professor, Division of Periodontology, Department of Oral Interdisciplinary Medicine, Graduate School of Dentistry, Kanagawa Dental University, Yokosuka, Japan. Assessed the feasibility of the experimental design.; e Associate Professor, Department of Highly Advanced Stomatology, Yokohama Clinic, Kanagawa Dental University, Kanagawa, Yokohama, Japan. Performed the statistical evaluation and contributed substantially to the discussion section of the manuscript.; f Professor, Department of Highly Advanced Stomatology, Yokohama Clinic, Kanagawa Dental University, Kanagawa, Yokohama, Japan. Performed the data analysis and interpretation; proofread the manuscript and performed the tests.

**Keywords:** tooth transplantation, complete root development, periodontal tissue healing

## Abstract

**Purpose:**

Tooth transplantation is a treatment that uses non-functional teeth to compensate for defects caused by tooth extractions. Surgical procedures have yielded high success rates in autologous tooth transplantation using a tooth with a complete root. This study aimed to evaluate periodontal tissue healing after transplantation of 14 molar teeth.

**Materials and Methods:**

Fourteen individuals aged 28–53 years who underwent autologous transplantation of third molars with completely developed roots between December 2010 and March 2017 were included in the study. The donor tooth was carefully extracted, placed into the prepared transplant site, and stabilised with an orthodontic wire and 4-0 silk sutures for a few weeks. Endodontic treatment was performed after 3–4 weeks. To evaluate the periodontal tissue healing, clinical measurements of the probing pocket depth (PPD), clinical attachment level (CAL), and keratinised gingival width (KGW) were performed, along with radiographic examinations of bone defect fill (BDF) at baseline and at 6 and 12 months after surgery. Statistical analysis was performed using the Mann–Whitney U test.

**Results:**

The changes in PPD and CAL at baseline, 6, and 12 months were statistically significant (*P* <0.05). KGW did not show a statistically significant decrease. The postoperative-BDF amount at 6 and 12 months was 2.2 ± 1.4 and 4.2 ± 1.4 mm, respectively.

**Conclusion:**

Periodontal tissue healing may occur in tooth autotransplantation even in the presence of complete root development in the donor tooth.

Several multicentre randomised controlled trials and case–control studies have reported tooth autotransplantation to be a viable intervention for a missing tooth. Numerous clinical studies on this topic have been conducted since the 1970s; however, most have reported transplantations of teeth with incompletely developed roots. These studies focused on factors such as donor tooth development, eruption stages, root growth, pulp healing and root resorption.^6, 14^

Moreover, the loss of transplanted teeth due to root resorption caused by dental pulp necrosis and damage to the periodontal ligament (PDL) during transplantation has been reported.^5^ The reportedly high success rate of transplanted teeth with completely developed roots is likely due to improved surgical procedures involving root canal treatment and prevention of PDL damage.

Tsukiboshi evaluated 250 cases of autologous tooth transplantation in extraction sockets and reported a survival rate of 100% and a success rate of approximately 95% over 6 years.^20^ Lundberg et al reported a higher success rate for autotransplantation of teeth with developing roots (94.1%), compared to that of completely developed roots (83.7%).^12^ In contrast, Majare et al reported a success rate of 95% over 2 years for teeth with completely developed roots.^13^

Currently, tooth autotransplantations are less successful than dental implants due to complications such as external root resorption and periodontal pocket formation.^17, 18^ However, autotransplantations have shown aesthetic benefits such as maintenance of the natural form of the attached gingiva; further, it also facilitates favourable periodontal wound healing and maintenance of the alveolar bone and prevents tooth movement through preservation of the PDL.^10^

Although there are studies that have reviewed and shown the long-term prognosis of tooth transplantation, there have been very few studies reporting the periodontal healing process around the transplanted tooth.^16^ Therefore, this retrospective study aimed to clinically and radiologically evaluate periodontal tissue healing following autologous transplantation of 14 molars with developed roots.

## Materials and Methods

This study was conducted in accordance with the ethical principles outlined in the Declaration of Helsinki (2013) and approved by the ethics committee of the authors’ affiliated institution. This study was approved by the Ethics Committee of XXX Dental University on 29 March 2018. This study was retrospectively registered in XXX Dental University Clinical Trials Registry (ID:486). Written informed consent was obtained from all participants after treatment. The inclusion criteria were as follows: age >20 years; absence of advanced periodontal disease; absence of diabetes, heart disease, pregnancy, or oral malignancy; preoperative plaque control and bleeding on probing <20%; follow-up period of over 1 year; and unimpacted third molar. All patients provided written informed consent. Fourteen patients (7 men and 7 women; average age, 37.8 years; range, 28–53 years) who underwent autologous transplantation of third molars with completely developed roots at our university between December 2010 and March 2017 were included in the study. The 14 transplanted teeth were retrospectively examined. None of the participants had systemic illnesses that could have led to infections and/or affect soft tissue healing or bone formation, and there were no systemic or local contraindications to the performed surgical procedures.

All participants received an initial treatment comprising oral hygiene instructions, plaque removal, curettage of periodontal pockets, caries removal, and correction of occlusal disturbances.

**Fig 1:**
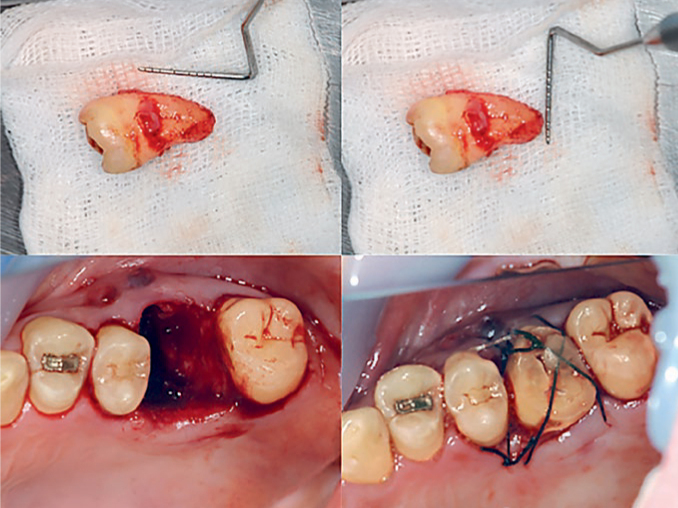
Representative clinical photographs demonstrating the surgical procedure.

All assessments were performed by experienced, trained examiners (AT and TS), excluding the surgeon (YK). All patients were treated under local anaesthesia (2% lidocaine with epinephrine, 1:80,000). Orthopantomography was performed before the surgery to confirm the position of the third molar and its root development. Orthopantomography was used to evaluate the recipient site, root development and the position of the donor tooth. However, it was difficult to determine the details of the alveolar bone, root resorption and the presence of the PDL using orthopantomography. The surgical protocol followed was according to that described by Andreasen et al.^2^ Precautions were taken to avoid PDL damage, and the donor tooth was carefully removed. For the recipient site, the root length and width of the extracted tooth were measured using a periodontal probe, and the recipient site was formed using a round bar and a trephine bar to form a socket with the same root length and apical width (Fig 1). While preparing the recipient site, the tooth was stored in a sterile saline solution. The donor tooth was placed in the prepared transplant site, and the flap was sutured with careful adjustment to the morphology of the crown of the transplanted tooth. The transplanted tooth was positioned slightly below the occlusal plane and stabilised with an orthodontic wire and 4-0 silk sutures. All patients received antibiotic coverage with amoxicillin for 4 days (daily dose of 750 mg), combined with prescription anti-inflammatory drugs. Additionally, patients were instructed to use 7% povidone–iodine mouthwash three times a day for 1 week. The sutures were removed after 1 week, and the fixation 3 weeks postoperatively. Endodontic treatment was performed by an endodontist postoperatively following 3–4 weeks. The treatment included debridement of the root canal system, followed by a temporary filling of the root canals with calcium hydroxide, which was changed every 4 weeks until 6 months after surgery. The condition of the root canals was monitored using radiographs. All teeth were obturated with thermoplasticised gutta-percha using the lateral condensation technique; there were no signs of failure clinically or radiographically. The restorative and prosthetic treatments were completed either by the dentists who had encouraged the patients to undergo the tooth transplantation, or by the dentists at our clinic. Clinical and radiographic examinations were performed at baseline, and at 6 and 12 months after surgery.

Clinical measurements of the experimental sites were performed immediately (baseline), and 6 and 12 months after tooth transplantation. At baseline, the measurements of the splinted teeth were performed at the experimental sites. The probing pocket depth (PPD) and clinical attachment level (CAL) were measured by trained examiners using a periodontal probe. Probing measurements were performed by two calibrated examiners (AT and TS) using a manual pressure-sensitive probe (at approximately 0.25 N force) with 1 mm increments (Gram Probe #2, YDM, Tokyo, Japan). Measurements were approximated to the nearest millimetre. CAL was defined using the cementoenamel junction (CEJ) as reference. If an interproximal restoration was present, the apical-most extension of the restoration (restoration margin) was used as a reference to measure the CAL. The intraosseous defect in this study was formed by the fixed tooth transplantation and residual alveolar bone at baseline. These were measured at six sites (mesiobuccal, mesiolingual, lingual, distolingual, distobuccal, and buccal) on the teeth. The deepest sites in the selected defects were recorded. The keratinised gingival width (KGW) was measured from the buccal central gingival margin to the mucogingival junction, which was stained with iodine. Tooth mobility was evaluated using the Miller classification (1950). Standardised intraoral radiographs were obtained at baseline and at 6 and 12 months after surgery.^15^ The linear distance measurements were performed on digital radiographs according to the method described by the digital radiograph processing system Cross Tech (Cross Tech Corporation, Tokyo, Japan), and Tonetti et al^19^ and Falk et al.^7^

Dimensions of the intraosseous defects were measured from the CEJ or the restoration margin to the alveolar bone crest, and to the bottom of the defect using the baseline radiographs. These dimensions were measured at the deepest interdental point of the defect. The width of the intraosseous defect was defined as the vertical distance from the coronal point of the alveolar crest to the root surface (mesiodistal), and the length of the buccolingual (palatal) defect. The depths of the one-walled, two-walled, and three-walled defects were converted to the nearest millimetre, and the depth of the intraosseous defect was derived. The linear distances were evaluated as follows: distance from the CEJ or the restoration margin to the root apex area (CEJ–RAA); distance from the CEJ to the top of the interdental bone crest (CEJ–TBC); and distance from the CEJ to the bottom of the intraosseous defect where the PDL is visible with uniform width on the same root surface (CEJ–BID). The depth of the vertical defect was recorded as the distance from the top of the alveolar crest to the bottom of the intraosseous defect (TBC–BID). The bone defect fill (BDF) rate was calculated as BDF/TBC–BID × 100%. The CEJ–TBC at baseline and at 6 and 12 months after surgery was compared for the assessment of interdental bone resorption. The variability between baseline and post-treatment radiographs was calculated as the ratio between linear measurements of the CEJ–RAA at baseline and those at each time point after the treatment. The average ratio of the CEJ–RAA at each time point was 1.005 ± 0.104 (mean ± standard deviation). This ratio was used as the correction factor to estimate bone changes.

### Statistical Analysis

The clinical and radiographic measurements of the 14 teeth are described as mean ± standard deviation, before and after treatment. The statistical significance of differences in the clinical and radiographic measurements before and after treatment was analysed using the Mann–Whitney U test. The level of statistical significance was set at *P* <0.05. Statistical analysis was performed using the SAS software program (JMP ver. 11.2.0 2013, SAS institute, Tokyo, Japan).

## Results

**Fig 2:**
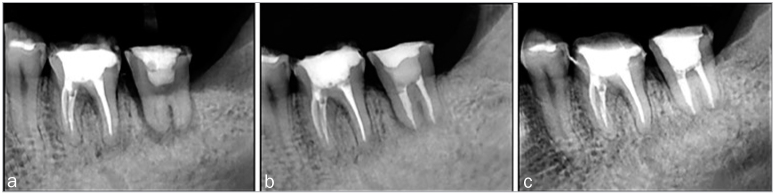
Conventional intraoral radiography. (a) 1 month after autotransplantation; (b) 6 months after autotransplantation; and (c) 12 months after autotransplantation.

**Fig 3:**
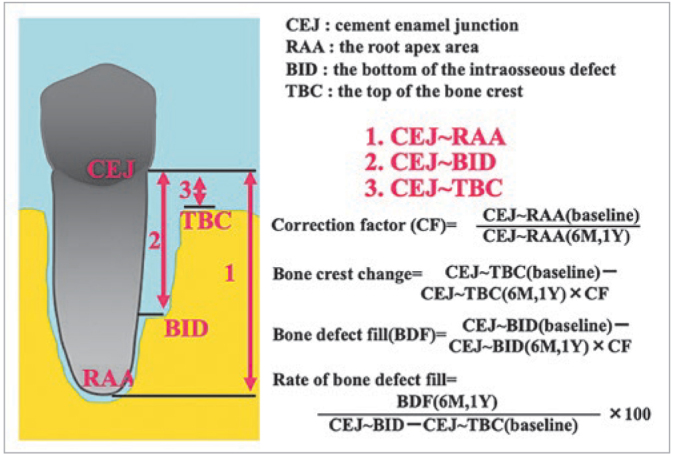
Measuring methods and measurement points on the digital radiograph (correction value of radiograph for root length, change of alveolar crest, intraosseous defect filling amount and rate).

**Fig 4:**
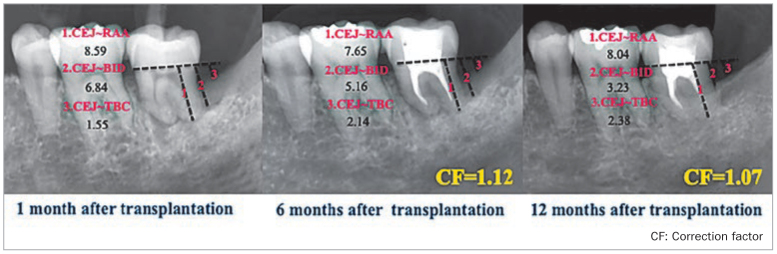
Actual measurements in a representative case (1 month, 6 months, 12 months).

**Fig 5:**
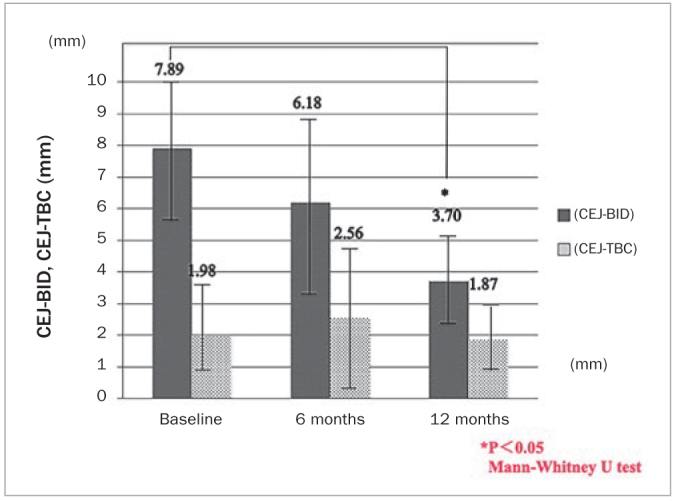
Changes in intraosseous defects and bone crest from the cementoenamel junction at 6 and 12 months postoperatively, relative to the baseline values.

**Fig 6:**
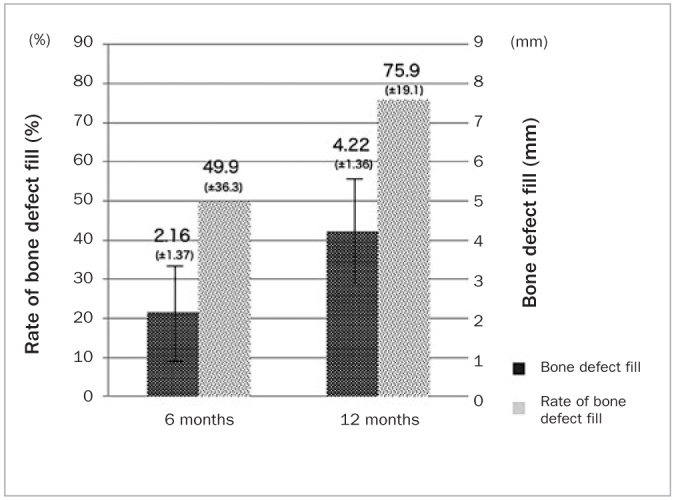
Comparative analysis of bone fill and the rate of bone fill at 6 and 12 months after autotransplantation.

Pain and tenderness decreased 2 weeks after autotransplantation of the tooth, and tooth mobility was graded as 1. One month after the transplantation, gingival morphology surrounding the transplanted tooth recovered sufficiently and appeared similar to the gingival morphology of the adjacent teeth. At 6 months, the mobility of the transplanted tooth had decreased to either 0 or 1, and the PPD was shallow. The radiographs did not show any radiolucency suggestive of tooth resorption. The marginal alveolar bone support was similar to that around the adjacent teeth. A nearly continuous PDL space was visible on the radiograph around the root of the transplanted tooth. The mean depth of the intraosseous defect was 7.9 ± 1.9 mm (Table 1). At 6 months, a CAL gain of 2.5 ± 1.0 mm was associated with a residual PPD of 3.4 ± 0.8 mm (2.2 ± 0.8 mm decrease in PPD). The changes in the PPD and CAL at baseline and at 6 and 12 months after tooth autotransplantation were statistically significant (*P* <0.05). At 12 months, a CAL gain of ≥ 2 mm was observed in all the patients. There was no statistically significant decrease in the KGW (Table 2). A representative case, the measurements at each time point, and the measurement methods are shown (Figs 2, 3 and 4). The radiographic measurement findings show that the distance from the CEJ to the BID was reduced at 6 months, compared with the baseline (6.1 ± 2.5 mm, range, 1.6–10.4 mm). This reduction was statistically significant at 12 months (3.7 ± 1.3 mm, range 1.2–7.0 mm) (*P* <0.05). There was no statistically significant difference in the distance from the CEJ to the TBC at baseline, and 6, and 12 months after the surgery (Fig 5). At 6 and 12 months, the BDF amount was 2.2 ± 1.4 mm (range, 0.3–5.1 mm) and 4.2 ± 1.4 mm (range, 1.6–6.5 mm), respectively, and the BDF rate was 49.9% ± 35.0% (range, 4.0–141.0 %) and 75.9% ± 16.9% (range, 59.7–108.3%), respectively (Fig 6). At 12 months, the BDF rate was ≥ 50% in all cases and ≥ 80% at the five marked sites.

**Table 1:** Intraosseous defect characteristics of around tooth autotransplantation

	Two- or three-walled (mean ± SD)	One-walled (mean ± SD)	Total (mean ± SD)
No. of patients	11	3	14
Defect depth	7.7 ± 2.1 mm	8.5 ± 0.4 mm	7.9 ± 1.9 mm
Mesiodistal defect width	2.8 ± 1.1 mm	5.3 ± 2.5 mm	3.3 ± 1.7 mm
Buccolingual defect width	5.5 ± 1.7 mm	9.3 ± 0.5 mm	6.4 ± 2.2 mm

SD: standard deviation.

**Table 2:** Changes in pocket probing depth, clinical attachment level, tooth mobility and keratinised gingival width after tooth autotransplantation in 14 patients

	Baseline (mean ± SD)	6 months (mean ± SD)	12 months (mean ± SD)
PPD	5.6 ± 0.8 mm	3.4 ± 0.8 mm*	2.9 ± 0.8 mm*
PPD reduction from baseline		2.2 ± 0.8 mm	2.8 ± 7.9 mm
CAL	6.5 ± 1.8 mm	3.9 ± 1.7 mm*	3.3 ± 1.2 mm*
CAL gain from baseline		2.5 ± 1.0 mm	3.2 ± 0.9 mm
Mobility	1	0	0
KGW	2.9 ± 0.6 mm	2.4 ± 0.8 mm	2.2 ± 0.7 mm

*Statistically significant difference from baseline (*P* <0.05, Mann–Whitney U test) PPD: probing pocket depth, CAL: clinical attachment level, KGW: keratinised gingival width.

## Discussion

The success rate of all participants in this study was 100%, which was consistent with that reported previously. In this study, we clarified the distribution of maxillary and mandibular third molars and the location of recipient sites (Table 3). The success rate was 100%, which is considered to be related to the same jaw and same part; in our study, this was 11 of 14 cases. Therefore, our findings demonstrated that there is little relationship between the differences in anatomical factors. However, to our knowledge, no author has documented the process of favourable periodontal wound healing using the parameters analysed in our study. We aimed to explore the efficacy of periodontal wound healing in tooth autotransplantation. The most statistically significant factor for successful tooth autotransplantation is the PDL function associated with the transplanted tooth.^2^ The PDL is responsive to pH and osmotic pressure changes, and its function is affected if extraoral dry time is prolonged.^4^ Previous research has reported that the survival rate of PDL exposed to an environment outside the oral cavity deteriorates rapidly after approximately 18 min.^11^ In our case series, the teeth to be transplanted were stored in sterile saline-moist gauze during the preparation of the recipient site, and the transplant was completed in less than 30 min in each patient.

**Table 3:** The distribution of the maxillary and mandibular third molars and the location of the recipient sites

Recipient site	Donor site	Cases
Maxillary left second molar	Maxillary left third molar	4
Mandibular left second molar	Mandibular left third molar	4
Maxillary right second molar	Maxillary right third molar	2
Mandibular right second molar	Maxillary left third molar	1
Mandibular left second molar	Maxillary left third molar	1
Mandibular right second molar	Mandibular right third molar	1
Maxillary left first molar	Mandibular left third molar	1

**Fig 7:**
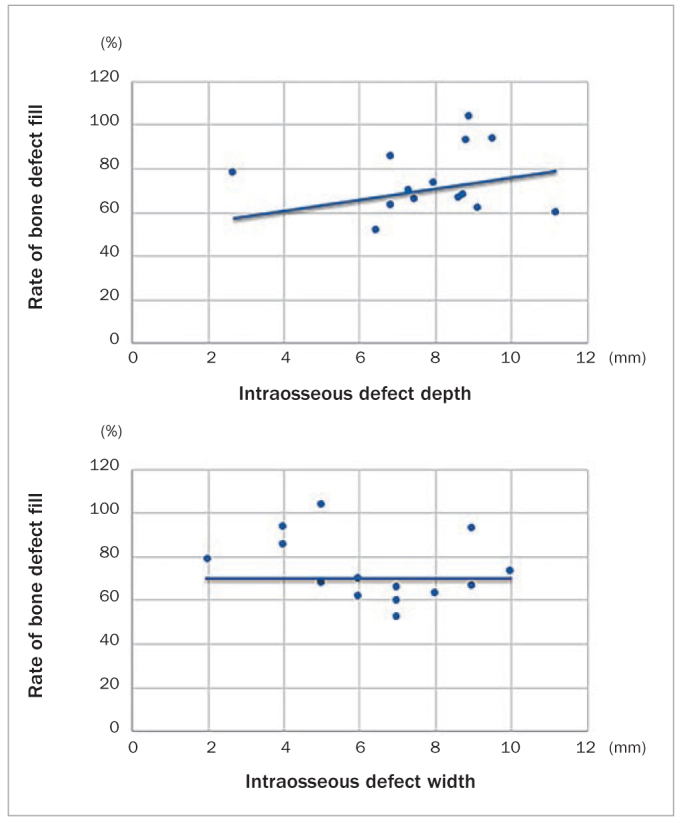
Correlation analysis of the bone defect fill rate and the intraosseous depth and width.

**Fig 8:**
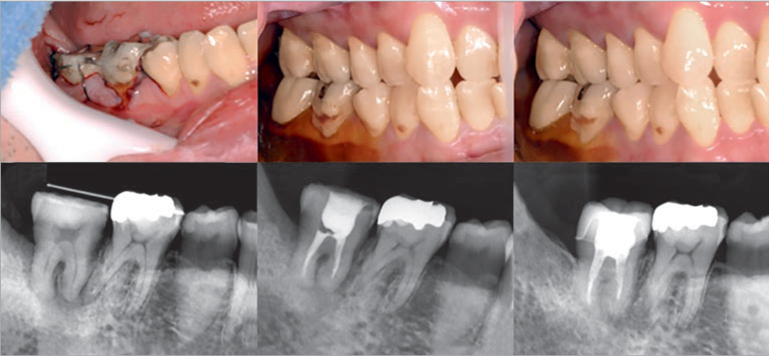
Clinical outcomes following an autologous tooth transplantation.

In this study, the baseline PPD that would affect the participants’ CAL increase was moderate to severe. Further, the results of the correlation analysis revealed that the radiographic-BDF rate positively correlated with the baseline intraosseous defect depth (7.9 ± 1.9 mm), but not with the baseline intraosseous defect width (6.4 ± 2.2 mm). Therefore, the amount of periodontal wound healing was maintained and did not decrease, even when the baseline intraosseous defect was wide (Table 1, Fig 7). Furthermore, the BDF rate improved to 49.9% ± 36.3% at 6 months and 75.9% ± 19.1% at 12 months. The decrease in PPD was 3.4 ± 0.8 mm at 6 months and 2.9 ± 0.8 mm at 12 months, and the increase in CAL was 3.9 ± 1.7 mm at 6 months and 3.3 ± 1.2 mm at 12 months (Table 2). Statistically significant improvement was observed, indicating good periodontal tissue healing (Fig 8). Moreover, the BDF associated with the transplantation resulted in bone regeneration of 2.2 mm and 4.2 mm relative to the defect 6 and 12 months after transplantation, respectively. This result indicates that periodontal wound healing associated with BDF after tooth autotransplantation is higher than that associated with the treatment of periodontal disease. We hypothesise that this may be because the intraosseous defect was very deep (up to 7.9 mm), and the PDL remained healthy and intact. The BDF after guided tissue regeneration and enamel matrix derivative graft is approximately 1 and 2 mm, 6 and 12 months after surgery, respectively.^3, 9^

Therefore, tooth autotransplantation is considered effective not only in cases with a wide deficiency range of the defect at baseline, but also in cases with excessive tooth mobility. Transplanted teeth may have poor outcomes if periodontal reattachment fails or if root resorption occurs at the engrafted cementum-root surface.^8^ Cementum reattachment failure may be induced by periodontal inflammation, alveolar socket inflammation or due to inadequate initial stabilisation after transplantation. Hence, transplantation is contraindicated in the presence of periapical infection. Furthermore, during tooth extraction, chronic inflammatory tissue must be completely removed.

In this study, the follow-up period was 13 months on average, which is slightly shorter than that of other studies; however, no inflammation or replacement root resorption occurred during this period. Our results are similar to the success rates of autotransplantation of teeth with a mature root apex as reported by Lundberg et al^12^ and Mejare et al.^13^ In our patients, no failed transplants were observed. Failure may also be attributed to poor periodontal conditions, additionally aggravated by incomplete removal of chronic inflammatory tissue. Other possible reasons include loss of initial fixation and increased tooth mobility.

In conclusion, tooth autotransplantation is performed in patients in whom rehabilitation is impossible due to factors such as advanced dental caries, root fracture and/or failure of endodontic treatment. It involves transplantation and stabilisation of the patient’s tooth into the socket of a missing tooth. With proper management of the case, KGW is maintained and the success rate is relatively high, greatly contributing to the prolonged function of natural teeth.^1^ Finally, tooth autotransplantation provides favourable outcomes in terms of healing responses in the hard and soft tissues (ie, increase in CAL, pocket reduction, maintenance of the keratinised gingiva, and bone fill).
